# Anatomical site-specific contributions of pneumococcal virulence determinants

**DOI:** 10.1186/s41479-016-0007-9

**Published:** 2016-06-03

**Authors:** Anukul T. Shenoy, Carlos J. Orihuela

**Affiliations:** Department of Microbiology, The University of Alabama at Birmingham, Bevill Biomedical Research Building, Room 669 845, 19th Street, South Birmingham, AL 35294-2170 USA

**Keywords:** *Streptococcus pneumoniae*, Pneumococcus, Pathogenesis, Virulence, Host-pathogen interactions

## Abstract

*Streptococcus pneumoniae* is an opportunistic pathogen globally associated with significant morbidity and mortality. It is capable of causing a wide range of diseases including sinusitis, conjunctivitis, otitis media, pneumonia, bacteraemia, sepsis, and meningitis. While its capsular polysaccharide is indispensible for invasive disease, and opsonising antibodies against the capsule are the basis for the current vaccines, a long history of biomedical research indicates that other components of this Gram-positive bacterium are also critical for virulence. Herein we review the contribution of pneumococcal virulence determinants to survival and persistence in the context of distinct anatomical sites. We discuss how these determinants allow the pneumococcus to evade mucociliary clearance during colonisation, establish lower respiratory tract infection, resist complement deposition and opsonophagocytosis in the bloodstream, and invade secondary tissues such as the central nervous system leading to meningitis. We do so in a manner that highlights both the critical role of the capsular polysaccharide and the accompanying and necessary protein determinants. Understanding the complex interplay between host and pathogen is necessary to find new ways to prevent pneumococcal infection. This review is an attempt to do so with consideration for the latest research findings.

## Background

*Streptococcus pneumoniae* (pneumococcus) is a Gram-positive, lancet-shaped bacterium that has diplococci morphology, is typically encapsulated, and is non-motile. In most instances *S. pneumoniae* resides asymptomatically in the nasopharynx of healthy individuals [1]. Yet this opportunistic pathogen is associated with devastating morbidity and mortality in vulnerable populations such as young children, the elderly, and those who are immunocompromised [2, 3]. *S. pneumoniae* is capable of causing a myriad of diseases including sinusitis, conjunctivitis, otitis media, and pneumonia, also invasive diseases such as bacteraemia, sepsis, and meningitis [1, 2]. Worldwide, it is the leading cause of death in young children and of infectious death in the elderly [3, 4]. Although the incidence of disease that develops in carriers is generally low, the vast numbers of colonised individuals make *S. pneumoniae* a major burden with significant socio-economic costs. For all these reasons, efforts to create a viable vaccine against *S. pneumoniae* date back as far as 1911 [5].

*S. pneumoniae* virulence determinants can be divided into 3 categories: capsule, cytotoxic products, and surface proteins. The extracellular capsule is a structure of complex sugars that surround the bacteria and form a protective barrier. On the basis of the biochemical composition and the serology of the polysaccharide, pneumococci are classified into 97 distinct capsular serotypes [6]. The capsule allows the pneumococcus to evade mucociliary clearance, complement deposition, and opsonophagocytosis [7, 8]. A critical role for the capsule is highlighted by the fact that antibodies specific to a capsule type are highly protective against invasive pneumococcal disease by strains belonging to the same serotype [9, 10]. As such, development of antibodies against the capsule is the basis of the current vaccines that are composed of polysaccharides conjugated to protein, and the older vaccine formulations that were composed solely of purified capsular polysaccharides [11]. Importantly, extensive epidemiological evidence suggests that pneumococci belonging to different serotypes vary in their prevalence and propensity to cause invasive disease. Isolates belonging to serotypes 6A, 6B, 19F, and 23F were found to be more prevalent colonisers of children younger than 5 years of age, while isolates belonging to serotypes 3, 9, and 23F were more common in adolescents and adults before the introduction of the first conjugate vaccine [12, 13]. On the contrary, serotypes 1, 4, 5, and 7F (which are known to be more invasive) colonise the population to a lesser degree [14, 15]. The current conjugated vaccines are composed of the polysaccharides that are most commonly carried by strains that cause the bulk of disease in humans.

It is important to note that the introduction of the 7-valent pneumococcal conjugate vaccine (PCV7) covering serotypes 4, 6B, 9 V, 14 18C, 19F, and 23F in the year 2000 reduced the incidence of invasive pneumococcal disease (IPD) in children of countries that implemented the vaccine [10, 16–19]. Yet, PCV7 had only a modest effect in reducing the incidence of otitis media caused by the PCV7-covered pneumococcal serotypes [20]. Moreover, there has been a rise in the incidences of infections caused by non-PCV7-covered serotypes [21, 22], a phenomenon known as serotype replacement. To address this problem, a 13-valent pneumococcal conjugate vaccine (PCV13) covering 6 additional serotypes (1, 3, 5, 6A, 7F, and 19A) was introduced in 2010. Despite the elevated effectiveness of PCV13, reports of continued serotype replacement by non-PCV13 pneumococcal serotypes have been documented [23, 24]. Recently, a 15-valent pneumococcal conjugate vaccine containing the serotypes in PCV13 and an additional 2 serotypes (22F and 33F) has been developed to address this shift and further curb pneumococcal carriage and invasive disease [25]. Importantly, recent epidemiological studies suggest there is also a rise in the number of individuals colonised with non-encapsulated *S. pneumoniae* [26]. This is presumably driven by both vaccine and antibiotic selective pressures, although increased sensitivity in our detection methods may account for increasing numbers. The importance of these non-encapsulated strains to human health is an open question. What is more, their impact may be indirect, for example they may act as a reservoir of antibiotic resistance genes for encapsulated strains [27].

The capsule by itself cannot be signed off as the sole virulence determinant responsible for human disease. More than 60 years of evidence suggests that the toxin pneumolysin (Ply) and diverse surface proteins are involved in tissue damage, modulation of the host response, immune evasion, and adhesion and invasion of cells and tissues [28, 29]. This requirement for non-capsular determinants is reinforced by observations that show: (i) isogenic capsule serotype-switching does not always confer virulence [30]; (ii) clinical isolates of *S. pneumoniae* belonging to the same serotype vary in their ability to cause disease [31]; and (iii) isogenic deletion of protein determinants can drastically attenuate the ability of *S. pneumoniae* to progress from one anatomical site to the next [32]. When considering all of these factors together, one must conclude that pneumococcal virulence is the result of the right combination of capsule, cytotoxic factors produced, and surface protein virulence determinants.

This review will discuss how different pneumococcal determinants impact the disease progression in an anatomically site-specific manner. To understand the mechanisms of pneumococcal evasion of the immune system it is necessary to understand anti-pneumococcal mechanisms of innate and adaptive immunity in the host.

## Pneumococcal colonisation of the respiratory mucosa

### The role of the pneumococcal capsule in persistence within the respiratory mucosa

*S. pneumoniae* transmission between individuals occurs via aerosolised droplets that are inhaled and via pneumococcus contaminated fomites that introduce the bacteria to the oropharynx (e.g., saliva coated toys in daycare settings). As such, the dominant mechanisms of defense against the pneumococcus include the physical barrier formed by the mucosal surface of the respiratory tract, soluble antibacterial components present in the mucus, and the innate and adaptive immune cells residing in the mucosal linings. To counter bacterial adherence and colonisation of the nasopharyngeal epithelia, the negatively charged mucus layer of epithelial lining forms the first line of defense. Pneumococci have evolved different degrees of negatively charged capsules to evade entrapment and mucus-dependent clearance [8]. With the exception of serotypes 7A, 7F, 14, 33F, and 37, all pneumococcal capsules with known biochemical structure possess a net negative charge [33]. The capsular negative charge electrostatically repels phagocytic macrophages and neutrophils that are also negatively charged, and sterically inhibits receptor ligand interactions with pneumococcal surface components [34, 35]. The importance of the net capsular charge is best evidenced by the observation that the serotypes that possess the greatest negative charge are those that exhibit the highest carriage prevalence in human populations [34].

### Resistance to anti-microbial secretions

The respiratory tract epithelial lining secretes lactoferrin, various cationic antimicrobial peptides (AMPs), and lysozyme with anti-pneumococcal activity [36, 37]. Lactoferrin sequesters the iron necessary for bacterial metabolism, hence exerting its antimicrobial activity [36]. Lysozyme enzymatically hydrolyses the conserved β-1,4-glycosidic bonds between N-acetylglucosamine and N-acetylmuramic acid, the disaccharide building blocks of the peptidoglycan backbone of Gram-positive bacteria. To evade cationic AMP-mediated killing, *S. pneumoniae* has been shown to incorporate D-alanine in its cell wall techoic acid component to reduce cell surface negative charge [38]. Most pneumococci express the serine protease PrtA that cleaves human apolactoferrin (the iron-free form of lactoferrin). This yields a very potent bactericide called lactoferricin. Clones of *S. pneumoniae* capsular serotypes 2 and 19F strains with mutations in the *prtA* gene, have demonstrated a loss of their ability to convert apolactoferrin to lactoferricin rendering them resistant to lactoferrin killing [37]. Pneumococcal surface protein A (PspA), a choline-binding protein, binds to the active site of apolactoferrin through electrostatic interactions thus sequestering it and blocking apolactoferrin-mediated bacterial killing [39, 40]. Two enzymes—PdgA, a N-acetylglucosamine deacetylase and Adr, an O-acetyl transferase—have been shown to modify the pneumococcal cell wall making it resistant to lysozyme-mediated degradation [41].

Nasopharyngeal secretions are also rich in secretory immunoglobulin A (sIgA), which bind to and aggregate pneumococci [42]. This facilitates their opsonisation and promotes phagocytosis; it also anchors the bacteria in the mucus for mechanical clearance [43]. Pneumococci have evolved an IgA1 protease enzyme, which cleaves the sIgA at its hinge region abrogating the sIgA-mediated aggregation, thus allowing individual pneumococci to block their aggregation and entrapment within mucous and gain access and colonise the underlying nasopharyngeal mucosa [44].

### Pneumococcal adhesins during colonisation

While the capsule is required for virulence, it also negatively impacts the ability of pneumococci to adhere to mucosal epithelial cells [45]. This has been attributed to net charge as well as the reduced opportunity of bacterial adhesins to interact with their ligands on the host cell surface. To address this, pneumococci have been demonstrated to alter capsule expression levels in response to environmental cues such as oxygen availability, as well as in a stochastic manner via phase variation [46]. Pneumococci within the nasopharynx of colonised animals tend to belong to the transparent phenotype whereby they express lower levels of capsule and higher levels of certain cell surface adhesins like choline-binding protein A (CbpA) [46, 47]. In contrast, pneumococci isolated from the blood tend to belong to the opaque phenotype (and express more capsule), which is an inhibitor to adhesion but protects against opsonophagocytic killing [48]. The role of phenotype switching is discussed in greater detail below.

The ability to adhere to host cells results from the collective contribution of diverse surface proteins that include choline-binding proteins and microbial surface components recognising adhesive matrix molecules (MSCRAMMS) [49]. CbpA, also referred to as PspC, binds to the polymeric immunoglobulin receptor (pIgR) and laminin receptor (LR), while pneumococcal cell wall phosphorylcholine (ChoP) binds to platelet-activating factor receptor (PAFR) on the host cell surface allowing attachment and subsequent transcytosis to breach the host respiratory epithelial barrier [42, 50–52]. Other choline-binding proteins play roles in adhesion but also evasion of the host defense (discussed below), autolysis, fratricide, and decoration of the cell surface with ChoP [53, 54]. Mutants deficient in these proteins have been demonstrated to be less fit and avirulent in comparison to the wild type [55].

The extracellular matrix components present in the airways also serve as attachment ligands for pneumococci. These are targeted by MSCRAMMS. For example, pili are multi-subunit surface structures that enhance pneumococcal adherence to collagen I, fibronectin, and laminin [56]. Pilus islet-1 (PI-1) encodes RrgA, RrgB, and RrgC, which together make up the stalk and tip of the pilus in pneumococci [56, 57]. The adhesive pili tip, composed of RrgA, mediates binding to the host respiratory epithelium and modulates tissue invasion [56, 58]. Certain *S. pneumoniae* serotypes, for example serotypes 1, 2, 7 F, 19A and 19F, which are considered to be the emerging serotypes in industrialised and developing countries, have been found to express an alternative pilus islet-2 (PI-2) [59]. PI-2 further enhances adherence to host cells [59]. Pneumococcal adhesion and virulence proteins PavA and PavB are also MSCRAMMs that bind to fibronectin glycoproteins on epithelial cells facilitating nasopharyngeal colonisation and persistence [60, 61]. Bound extracellular matrix components not only serve as anchors for the pneumococcus in the nasopharynx, but also presumably as bridging molecules between pneumococci, thereby allowing for bacterial microcolonies or biofilms to develop [62].

*S. pneumoniae* also expresses carbohydrate-active enzymes (CAZymes) such as neuraminidase A (NanA), beta-galactosidase (BgaA), and beta-N-acetylgucosaminidase (StrH) that can modify a wide range of host glycans in the mucus, liquefying it to prevent entrapment [63, 64]. These exoglycosidases also liberate terminal monosaccharides such as sialic acid, galactose and N-acetylglucosamine, respectively, from the host cell surface. This is thought to promote the adherence of *S. pneumoniae* by exposing cryptic binding sites (e.g., N-Acetyl-D-Galactosamine) on the epithelial surface and the released monosaccharides serve as a carbon source to sustain growth [63, 65]. To further aid effective colonisation, pneumococci have also been implicated in inducing actin cytoskeleton disorganisation and disrupting the respiratory epithelial architecture, resulting in a loss of efficient mucociliary clearance [66]. Ply has also been shown to inhibit ciliary function and cause considerable cell damage [67]. Other virulence factors such as hydrogen peroxide produced by SpxB (pneumococcal pyruvate oxidase), hyaluronate lyase, and secreted metalloproteases augment the Ply-mediated injury to the human respiratory epithelium [68, 69]. Tissue inflammation increases the expression of known pneumococcal ligands such as pIgR, LR, and PAFR [70]. A new and important adhesin is PspK. The gene encoding PspK is found in place of the capsule operon. PspK has been implicated in otitis media caused by unencapsulated *S. pneumoniae* [71, 72]. Other proteins, such as enolase, that are normally thought to be intracellular housekeeping proteins, have been demonstrated to have important adhesin and immune system evading properties [73, 74]. A comprehensive review focused on pneumococcal adhesins and their possible use as novel vaccine antigens is available [75].

### Pneumococcal biofilms

Persistent nasopharyngeal colonisation and middle ear infection by *S. pneumoniae* is now known to involve biofilms [76, 77]. Biofilms are surface attached microbial communities encased within an extracellular matrix made up of complex carbohydrates, proteins and nucleic acids [78]. In vitro and in vivo *S. pneumoniae* within biofilms are predominantly in the transparent phenotype with a low-capsule and high cell wall techoic acid expression aiding in strong adhesion properties to host cells [46, 79]. Numerous pneumococcal proteins including NanA, LytA and SpxB have been associated with biofilm formation [80, 81]. In vitro, hydrogen peroxide produced by SpxB induces mutations in the capsule-encoding operon, resulting in pneumococcal variants with little to no capsule [82]. Whether this occurs in vivo is not known. SpxB-deficient mutants are, however, more encapsulated than wild-type *S. pneumoniae* [83]. As indicated earlier, transparent pneumococci in biofilms express elevated levels of certain adhesins such as CbpA, factors that modify host surface such as NanA, and lower levels of Ply [47]. The pneumococcal serine-rich repeat protein (PsrP) has also been documented to be up-regulated in biofilms [47]. PsrP permits pneumococcal adhesion to cytokeratin 10 on lung epithelial cells but also mediates intra-species pneumococcal tethering that facilitates the formation of biofilms [84, 85]. The fact that pneumococci colonising the nasopharynx asymptomatically (presumably within biofilms) are less capsulated with a higher-level expression of adhesin proteins suggests a very complicated interplay between various pneumococcal virulence determinants, one that is only in part mediated by phase variation. Of note, considerable evidence suggests that pneumococci within biofilms modulate virulence gene expression and are less invasive than their planktonic counterparts [47]. This suggests *S. pneumoniae* suppresses its virulence to promote asymptomatic carriage.

#### Otitis media

Otitis media or middle ear infection is one of the most common paediatric infections [12]. It is responsible for a tremendous socioeconomic burden in the form of hospital visits, surgical intervention, antibiotic therapy, and repeated infections may have long-lasting consequences such as hearing and speech impairment [86, 87]. One of the major difficulties with treatment of otitis media is the rising incidence of persistent and recurrent middle ear infection despite antibiotic therapy [88, 89]. *S. pneumoniae* is among the leading causes of such recurrent and persistent otitis media [90], with biofilm formation thought to play an important role [91, 92]. Although pneumococcal conjugate vaccines (PCV7/PCV13) have had considerable success in reducing the incidences of IPD, they have achieved only modest success with respect to the prevention of otitis media [93]. Epidemiological reports also suggest increasing prevalence of otitis media infections caused by non-encapsulated *S. pneumoniae* strains in vulnerable populations [26]. Antibiotic therapy is the most accepted mode of treatment for otitis media. However, persistent otitis media resistant to antimicrobial therapy is increasingly observed in humans and has been replicated as experimental models [94–96].

*S. pneumoniae* isolated from the middle ear of children with otitis media showed phase variation with a predominant tendency to possess an opaque phenotype, suggesting a different form of selection within the middle ear niche compared to the nasopharynx [97]. *S. pneumoniae* isolates from patients with otitis media were found to express MSCRAMMs such as pili (both PI-1 and PI-2), in addition to PspA, CbpA, and PcpA, and NanA [91, 98, 99]. Otitis media isolates showed enhanced adhesive abilities irrespective of their capsular serotype [99, 100]. Pneumococcal CAZymes such as NanA have also been shown to play a major role during middle ear biofilm formation wherein they liberate sialic acid residues from the tubotympanum of the experimentally challenged chinchillas and expose the underlying N-acetylglucosamine residues as potential attachment sites [101]. Pneumococcal autolysin and Ply have been implicated to play a role in pneumococcal pathogenesis of otitis media, which is characterised by neutrophil infiltration in the middle ear [102]. This Ply-mediated cytotoxicity is thought to explain the loss of outer hair cells within the ear cochlea and associated sensorineural hearing impairment following middle ear infection by *S. pneumoniae* [103]. Finally, it is now recognised that host innate and adaptive immune responses also play a major role in predisposing individuals to pneumococcal middle ear infection. Deficiencies in lysozyme M secretion, complement pathway activation, and mucosal antibody production have all been reported to increase susceptibility to otitis media [104–106]. In instances where otitis media is caused by unencapsulated *S. pneumoniae* strains, PspK seems to be a major determinant. In a chinchilla infection model, PspK has been shown to be important for progression of infection from the nasopharynx to middle ear [72].

### Host pathogen interactions involving innate immune mechanisms

The respiratory tract epithelium is home to resident macrophages and other immune cells that exert anti-bacterial activities [107]. Macrophages in the respiratory mucosa and monocytes and/or neutrophils that enter from the systemic circulation play the role of dominant effectors against pneumococcal infection, thus determining if the invading pneumococci can establish infection or are cleared without pathological consequences [108]. Clearance by macrophages is almost always effective; when this is not the case pneumonia develops and neutrophils are recruited to the airways.

One class of receptors that play an important role in macrophage surveillance against pneumococcal infection is scavenger receptors [109–111]. Scavenger receptors can function as pattern recognition receptors (PRRs) against bacterial pathogens and their activation induces a phagocytic response towards the microbes, even in the absence of their opsonisation [111]. The class A macrophage scavenger receptors (SRAs) can recognise the Gram-positive bacterial cell wall component lipotechoic acid and unmethylated CpG nucleotide sequences in bacterial DNA (CpG DNA) [112, 113]. They also function as co-receptors to Toll-like receptors (TLRs) such as TLR2 and Nod2, allowing a positive reinforcement to the innate inflammatory response to pneumococcal colonisation [111, 114]. SRAs (namely SRA-I/II and macrophage receptor with collagenous structure [MARCO]) expressed on the alveolar macrophages have been in implicated in anti-pneumococcal immunity [115, 116]. This was emphasised by increased susceptibility of mice lacking either of these receptors towards pneumococcal infection and associated pneumonic inflammation [109, 110].

Efficient pneumococcal clearance by macrophages first requires their activation in response to *S. pneumoniae* [117]. PRRs—such as TLR2, which recognises lipoproteins and lipotechoic acids in the pneumococcal cell wall; TLR4, which recognises Ply; TLR9, which recognises CpG DNA; and Nod2, which detects lysozyme digested peptidoglycans within the cytoplasm—have been found to contribute to macrophage activation and inflammatory response against pneumococci [118, 119]. Activation of macrophages via PRRs enhances phagocytosis, respiratory burst, major histocompatibility complex class II expression, and increases secretion of pro-inflammatory T helper (Th)1 and Th17 polarising cytokines like interleukin (IL)-8/killer cells (KC), granulocyte colony stimulating factor (G-CSF), macrophage chemoattractant protein-1, tumor necrosis factor alpha (TNF-α), interferon gamma (IFN-γ), IL-6, and IL-1α [120]. All these cytokines play a major role in orchestrating the host immune defense against pneumococci. While IL-8/KC and G-CSF are involved in neutrophil recruitment and activation, TNF-α and IFN-γ stimulates the Th1 and Th17 T cell lineages [121, 122]. IL-1α plays a vital role in T-cell expansion and survival [123, 124].

Ply also stimulates the Nod-like receptor NLRP3, thus activating the inflammasome complex, which results in secretion of the active forms of proinflammatory cytokines IL-1β, and IL-18 [125, 126]. Of note, clinical isolates of *S. pneumoniae* serotypes 1, 8, and 7F are associated with increased virulence despite reduced Ply activity due to their ability to evade NLRP3-mediated activation of the immune system [126]. Ply is also able to activate the classical complement cascade by binding to IgG, and in sufficient concentrations is able to kill cells directly [127]. The latter is through pore-formation-mediated loss of osmotic regulation, leading to cell lysis, but now also understood to be the result of both pyroptosis- and necroptosis-mediated killing at lower concentrations [128, 129]. Ply has also been implicated as being capable of killing phagocytic cells through rupture of the lysosome, once engulfed [130, 131]. Thus Ply is responsible for the subversion of the innate immune response against pneumococci by initiating macrophage, neutrophil and dendritic cell death [131, 132].

Successful nasopharyngeal colonisation by *S. pneumoniae* and Ply-mediated pore formation on epithelial cells exerts osmotic stress that activates p38 mitogen-activated protein kinase (p38 MAPK) signaling cascade [133]. This results in increased chemokine expression and the influx of neutrophils [8, 121]. Recruited neutrophils engulf and kill the bacteria by fusion of their antimicrobial granules with the phagosome. These phagosomes contain many reactive oxygen species and AMPs that can also be released by neutrophilic degranulation. Neutrophil-mediated killing is also enhanced by the pneumococcal capsule which sensitises the encapsulated bacteria to human neutrophil proteins 1 to 3 of the alpha defensin subfamily of AMPs exclusively produced by neutrophils [134, 135]. In addition to this, neutrophils on activation are also capable of releasing their chromatin DNA, which is bound to antimicrobial components like histones, elastase, myeloperoxidase, and lactoferrins that together form extracellular fibres called neutrophil extracellular traps (NETs) [136, 137]. Pneumococci trapped within the NETs escape using a membrane-bound surface endonuclease, EndA [138]. Pneumococci have also evolved alternative mechanisms for resistance to NET-mediated killing wherein they add D-alanine residues to their cell wall lipotechoic acid structure, thus gaining a positive charge that helps them electrostatically repel NET entrapment [139]. *S. pneumoniae* belonging to serotypes 1, 2, 4, and 9V possess capsules that further protect them against entrapment and killing mediated by NETs [139]. It is worth noting that excessive tissue inflammation and prolonged neutrophil infiltration is detrimental to the host and may permit the bacteria to escape from the lungs into the bloodstream [140, 141]. Thus neutrophils, despite being required for bacterial clearance, also enhance tissue destruction and indirectly facilitate pneumococcal dissemination into the circulation. The host must strike a careful balance between an insufficient and excessive immune response.

### Anti-pneumococcal adaptive immune mechanisms

Optimal host-mediated opsonophagocytic killing of *S. pneumoniae* involves both innate and adaptive immune mechanisms with complement and serotype-specific antibodies together providing the basis for strong anti-pneumococcal immunity. The fact that mice lacking CD8+ T cells were fully protected from pneumococcal challenge of any serotype but mice deficient in CD4+ T cells were not capable of clearing pneumococcal infection despite normal innate immune function highlights that the pneumococcus is an extracellular pathogen, and that there is a requirement for an adaptive response [142]. CD4+ T cells have been shown to migrate into the lungs early after intranasal challenge with *S. pneumoniae* in a Ply-dependent manner [143, 144]. Importantly, mice lacking 1L-17A receptors were not protected against subsequent pneumococcal colonisation, suggesting an important role for 1L-17A and thus the Th17 subset of CD4+ T cells in anti-pneumococcal acquired immunity [145]. IL-17A response to pneumococcal challenge efficiently recruits neutrophils and monocyte/macrophages into the airway lumen to further enhance the pneumococcal clearance [145, 146]. These observations were also recapitulated in experimental human pneumococcal carriage studies where carriage resulted in an increased number of Th17 cells in the airway lumen and enhancement in alveolar macrophage-mediated pneumococcal killing [147]. In addition to recruiting neutrophils via IL-17A, T cells have also been shown to modulate the antibody response against pneumococci forming an important link between the innate and adaptive immunity to *S. pneumoniae* [148, 149].

## Systemic pneumococcal infection

### Pneumococcal invasion of epithelial cells and endothelial cells

Pneumococci are capable of gaining entry into systemic circulation by translocation across respiratory epithelium and endothelial cells using two major mechanisms: intracellular migration and inter- or paracellular migration. ChoP acts as a molecular mimic of platelet-activating factor (PAF) and binds to PAFR on activated epithelial and endothelial cells, providing the bacterium with access via the PAFR recycling pathway [51]. This requires simultaneous interaction of CbpA with the LR [52], and allows pneumococcal migration to the basal membrane of host cell within vacuoles [51, 150]. Furthermore, CbpA also binds to the extracellular region of pIgR on the epithelial cell surface [42, 50]. This CbpA-pIgR interaction allows the pneumococcus to co-opt to the pIgR recycling pathway, and results in pneumococcal translocation within a vacuole towards the basal membrane of the epithelial cells [42, 151]. Of note, a study using 20 capsule-switched variants of *S. pneumoniae* TIGR4 strain found that successful pneumococcal invasion was highly dependent on the right combination of capsular serotype and CbpA, which together conferred resistance to complement recruitment and activation resultantly abrogating neutrophil opsonophagocytosis [152].

Inter- or paracellular migration of pneumococci across the epithelial and endothelial respiratory barrier can occur in two ways. Pneumococcus-bound plasminogen enhances pneumococcal adhesion to epithelial cells and endothelial cells, leading to cleavage of the intercellular cadherin junctions and allowing intercellular migration of pneumococci [153]. The recognition of pneumococcal cell wall lipotechoic acids by TLR2 is also shown to induce loss of epithelial barrier polarity by activation of p38 MAPK and transforming growth factor beta pathways, further promoting invasion [154]. Ply-mediated damage to the tissue barrier can also facilitate pneumococcal entry into the systemic circulation, enhancing invasion [67]. An important role for paracellular invasion has been demonstrated by the testing of type I interferon knockout mice that do not up-regulate tight junctions in response to bacterial infections. These mice developed bacteraemia at a rate considerably higher than their wild-type controls [155].

### Resistance to complement activation

Within the consolidated lungs and once pneumococci enter the bloodstream they are faced with a wide barrage of antibacterial host defense mechanisms. Typically, the innate immune mechanisms composed of the complement system, C-reactive proteins (CRPs), and phagocytic cells such as neutrophils and macrophages are considered to be of primary importance. The importance of complement systems in protection against pneumococcal infection is evidenced from the high susceptibility of mice and humans to pneumococcal infections when certain complement components are experimentally depleted or genetically deficient [156, 157]. Activation of the complement system involves recognition of specific molecular patterns on pathogens, a cascade of proteolytic cleavage involving several complement factors followed by bacterial killing, either by recruitment of the membrane attack complex or by opsonophagocytosis [158].

The complement system is comprised of 3 cascade pathways: the classical complement pathway, the alternative complement pathway, and the lectin-induced complement pathway. Naturally produced IgM specific against pneumococcal techoic acid and acute phase proteins (such as CRPs directed against ChoP in the pneumococcal cell wall) initiates complement subcomponent C1q deposition on the bacteria, inducing the classical complement pathway [159, 160]. The alternative complement pathway is induced against pneumococci by the direct interaction of the complement C3 with the pneumococcal cell wall, leading to C3b deposition on the bacterial surface [161, 162]. The lectin-induced complement pathway plays a less vital but still important role in protection against pneumococcal disease. Among the various lectins such as mannan-binding lectin, H-, L-, and M-ficolin, only L-ficolin and M-ficolin were found to activate the alternative complement cascade against the pneumococci. While L-ficolin bound to 3 capsulated *S. pneumoniae* serotypes (11A, 11D, and 11F) and ChoP moieties of the pneumococcal cell wall [163], M-ficolin bound to the extra N-acetylmannosamine residue linked via glycoside linkage within the capsules of 2 strains of *S. pneumoniae* serotype 19 (19B and 19C) [164]. Activation of the C1q by the classical or lectin-induced complement cascades leads to cleavage and activation of complement component C2, which then in combination with the activated fragment of C4 elicits C3b deposition [158]. Deposition of complements and CRPs enhances phagocytosis and induces cytokine production by immune cells [165, 166]. Splenic and liver resident macrophages have been reported to play a role in pneumococcal killing within systemic circulation and thus dampening the spread of bacteraemia [167].

The pneumococcal capsule blocks complement-mediated opsonophagocytosis by impairing the efficient binding of complement components on the bacterial surface, preventing proteolytic conversion of C3b to iC3b, and masking the cell-bound complements, thus hampering access of phagocytes to the opsonising complements [7]. The contribution of capsular serotypes in pneumococcal resistance to complements was reaffirmed when capsular serotype-switched *S. pneumoniae* TIGR4 background mutants showed different levels of susceptibility towards complement mediated opsonisation [168]. While capsular serotypes 6A and 23F strains on TIGR4 background showed more predisposition towards C3b/iC3b deposition and neutrophil phagocytosis, the capsular serotypes 4 and 7F strains on TIGR4 background showed resistance towards complement-mediated immunity and were more virulent in a mouse model of sepsis [168]. Additionally, pneumococci express a number of cell surface proteins that limit opsonisation-mediated killing. CbpA is capable of inhibiting opsonisation-mediated killing by binding and activating the complement regulatory protein factor H and thus inhibiting the alternative and lectin pathways [169], in addition to binding C4-binding protein and inhibiting the classical complement pathway [170]. However, the contribution of CbpA-mediated inhibition of complement cascade to pneumococcal infections is highly strain dependent. While CbpA deletion abrogated the virulence of *S. pneumoniae* serotype 4 strain TIGR4, the virulence of serotypes 2, 3, and 19F strains remained unaffected [171]. Isogenic capsule-switched strains on *S. pneumoniae* TIGR4 background showed large increases in deposition of factor C3b/iC3b on capsule-switched serotypes 4, 6A, 6B, and 9 V strains, but no significant difference in deposition on serotypes 2, 3, 17, and 23F strains [172]. This further bolstered the notion that pneumococcal virulence is mediated by a complex interplay between capsule and protein virulence determinants.

**Fig. 1:**
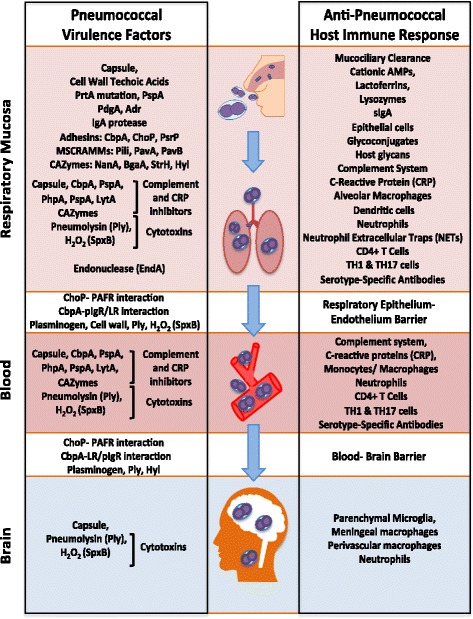
*Streptococcus pneumoniae* virulence determinants and host immune responses associated with pneumococcal infection in an anatomical site-specific manner. *S. pneumoniae* virulence factors known to play a major role in pneumococcal colonisation and infection of the respiratory mucosa, systemic circulation, and the brain, are listed in the left panel with the respective site-specific anti-pneumococcal host responses displayed in the right panel. The role of host–pathogen molecular interaction in pneumococcal migration across the respiratory epithelial–endothelial barrier and the blood–brain barrier is also highlighted in anatomical context. PrtA, serine protease; PspA, pneumococcal surface protein A; AMPs, antimicrobial peptides; PdgA, N-acetylglucosamine deacetylase; Adr, O-acetyl transferase; IgA, immunoglobulin A; CbpA, choline binding protein A; ChoP, cell wall phosphorylcholine; PsrP, pneumococcal serine rich repeat protein; MSCRAMMs, microbial surface components recognising adhesive matrix molecules; PavA, pneumococcal adhesion and virulence A; PavB, pneumococcal adhesion and virulence B; CAZymes, carbohydrate-active enzymes; NanA, neuraminidase; BgaA, beta-galactosidase; StrH, beta-N-acetylgucosaminidase; Hyl, hyaluronate lysase; PhpA, histidine triad protein A; LytA, pneumococcal autolysin; Ply, pneumolysin; SpxB, pneumococcal pyruvate oxidase; EndA, endonuclease; sIgA, secretory IgA; CRP, C-Reactive protein; NETs, neutrophil extracellular traps; PAFR, platelet activating factor receptor; pIgR, polymeric immunoglobulin receptor; LR, laminin receptor

PspA is a key pneumococcal surface protein that inhibits C3 binding on the surface of *S. pneumoniae*, avoiding complement-mediated opsonophagocytosis (Fig. 1) [173, 174]. PspA and PspC work synergistically to reduce this complement-mediated immune adherence and permit pneumococcal persistence in circulation [175] while PhpA is a surface-expressed protein capable of degrading C3 [176, 177]. As indicated previously, Ply is known to inhibit complement-mediated pneumococcal clearance by binding to the Fc portion of IgG, thus activating the classical complement pathway and sequestering complement factors away from the bacteria [178]. NanA, BgaA, and StrH have also been implicated in resistance to complement C3-mediated opsonophagocytosis in addition to their role in aiding pneumococci breach the nasopharyngeal epithelium barrier; this is via the deglycosylation of key host effector molecules [179].

### Resistance to CRP

CRP is an acute phase protein produced by the liver in response to IL-6 and other pro-inflammatory cytokines generated during systemic acute infection [180]. Epithelial cells in human respiratory tracts have also been reported to secrete CRP, thus serving as important players in the innate immune mechanisms against pneumococci [181]. During pneumococcal infections, CRP binds to ChoP on the pneumococcal cell wall resulting in the activation C1q [160]. PspA binds to ChoP moieties on the pneumococcal cell surface, competitively inhibiting the interaction of CRP with the cell wall [182]. Recent evidence suggests that the dominant pneumococcal autolysin LytA also prevents interaction of C1q and CRP, further reducing classical complement system activation [183]. In addition, LytA was also demonstrated to increase recruitment of complement inhibitors C4b-binding protein and factor H to the pneumococcal cell wall and actively degrade C3b and iC3b [183].

## Invasion of tissue from the bloodstream

### Pneumococcal meningitis

Systemic spread of *S. pneumoniae* within the circulation allows pneumococci access to the small blood vessels within organs. In the cranium, this provides access to, and can lead to translocation across, the blood–brain barrier into the subarachnoid space leading to bacterial meningitis. Worldwide, pneumococcal meningitis causes more than 50,000 deaths each year in children aged 5 years or younger. Those that survive often have long-term disabilities [184]. Pneumococci have been shown to translocate across the brain microvascular endothelial cells by binding to the vascular endothelial PAFR and LR, in a ChoP- and CbpA-dependent manner, similar to that previously described in pneumococcal invasion of epithelial cells [52, 185]. A recent study also reports that pIgR is present on the surface of brain microvascular endothelium; thus invasion may occur in this manner as well [186]. Importantly, adhesion to LR seems to have a conserved role in the pathogenesis of other neurotropic pathogens such as *Neisseria meningitidis*, and *Haemophilus influenzae* [52]. In addition to the intracellular translocation pathway, it is speculated that pneumococci also migrate across the vascular endothelial cell linings by disrupting the intercellular tight junctions during severe disease episodes. *S. pneumoniae* can bind and activate plasminogen in blood and cerebral spinal fluid, resulting in adhesion and damage to the extracellular matrix in in vitro models of bacterial meningitis and affected patients [187, 188]. Furthermore, pneumococci use hyaluronate lyase to digest various components of the intercellular milieu and host extra-cellular matrix, including hyaluronic acid, chondroitin and/or chondroitin sulfates to breach the blood–brain barrier [189]. Alternatively, it is thought that pneumococci may gain access through the sinuses and olfactory nerves following colonisation, in this instance causing occult meningitis (i.e., meningitis without airway or bloodstream infection) [190].

Once within the central nervous system, *S. pneumoniae* can cause damage to the brain microvascular endothelial cells during the course of pneumococcal meningitis [191]. Much of this ability to destroy the endothelial cell layer guarding the blood–brain barrier is attributed to pneumococcal Ply-mediated cytotoxicity in addition to the pneumococcal cell wall [192, 193]. The pneumococcal cell wall, and autolysis-mediated-Ply release induces a massive inflammatory response in the central nervous system [194]. Ply and hydrogen peroxide produced by the activity of SpxB contribute to the maximal neuronal apoptosis by caspase-dependent and independent mechanisms [195, 196]. Pneumococcal-mediated inflammatory damage incites the production of a wide array of cytokines that recruit immune cells to the site of pneumococcal infiltration. Neutrophils and other immune leukocytes have been found in spaces adjacent to subarachnoid arteries, meningeal veins, and also cerebral spinal fluid compartments like the subarachnoid spaces, meninges, and the corpus callosum of the brain in experimental meningitis models [197, 198]. Activated neutrophils secrete nitric and oxygen species [199], which may also contribute to neuronal damage, associated with pneumococcal meningitis.

### Pneumococcal cardio-invasion

Recently, our laboratory has reported that during invasive pneumococcal disease, *S. pneumoniae* circulating in the bloodstream are capable of invading the ventricular myocardium where they form bacteria-filled microscopic lesions (cardiac microlesions) [200]. These cardiac microlesions were accompanied by Ply-mediated cardiomyocyte death and aberrant cardiac functionality [200]. Similar to pneumococcal invasion of the blood–brain barrier, myocardial invasion was found to be dependent on ChoP-PAFR and CbpA-LR interaction [200]. The pneumococcal cell wall can bind to endothelial cells and cardiomyocytes in a PAFR-dependent manner, effectively exciting the cardiac vasculature and disrupting cardiac contractility, ultimately causing cell death [201]. Systemically circulating Ply also contributes towards the IPD-associated cardiac damage [202]. This IPD-associated cardiac damage might be responsible for the adverse cardiac events that occur in up to 25 % of all elderly patients suffering from community-acquired pneumonia [203, 204].

## Conclusion

This review highlights the various host–pathogen interactions associated with *S. pneumoniae* infection (Fig. 1). Understanding the basic pneumococcal biology and the complex link between its different virulence determinants will hopefully provide the insight necessary to solve the *S. pneumoniae* problem. Despite considerable variability known to exist between experimental challenge in animal models and pneumococcal infections of human populations, significant progress has been made towards this end. Further work and its translation to new and useful therapeutics is still required.

## Search strategy

The articles relevant to this review were identified by searching PubMed and Google Scholar for research papers and reviews (published in English only) including but not limited to “*Streptococcus pneumoniae*”, “pneumococci”, “pneumococcal”, “pneumonia”, “otitis media”, “virulence” and “pathogenesis”. To allow use of complete and detailed information no limits on date of publications were placed during the search. More suitable citations were further identified from the references in these initial searches.

## Abbreviations

Adr, an O-acetyl transferase; AMPs, antimicrobial peptides; BgaA, beta-galactosidase A; CAZymes, carbohydrate-active enzymes; CbpA/PspC, choline-binding protein A; ChoP, pneumococcal cell wall phosphorylcholine; CpG DNA, unmethylated CpG nucleotide sequences in bacterial DNA; CRPs, C-reactive proteins; EndA, pneumococcal surface endonuclease; G-CSF, granulocyte colony stimulating factor; Hyl, hyaluronate lyase; IFN-γ, interferon gamma; IgG, Immunoglobulin G; IgM, Immunoglobulin M; IL, interleukins; IPD, invasive pneumococcal disease; KC, killer cells; LR, laminin receptor; LytA, pneumococcal autolysin A; MARCO, macrophage receptor with collagenous structure; MSCRAMMS, microbial surface components recognising adhesive matrix molecules; NanA, neuraminidase A; NETs, neutrophil extracellular traps; NLRP3, NLR family, pyrin domain containing 3 protein; Nod2, Nucleotide-Binding Oligomerization Domain Containing 2; p38 MAPK, p38 mitogen-activated protein kinase signaling cascade; PAF, platelet-activating factor; PAFR, platelet-activating factor receptor; PavA, Pneumococcal adhesion and virulence protein A; PavB, Pneumococcal adhesion and virulence protein A; PcpA, Pneumococcal choline binding protein A; PCV13, 13-valent pneumococcal conjugate vaccine; PCV7, 7-valent pneumococcal conjugate vaccine; PdgA, N-acetylglucosamine deacetylase; PhpA, histidine triad protein A; PI-1, Pilus islet-1; PI-2, pilus islet-2; pIgR, polymeric immunoglobulin receptor; Ply, pneumolysin; PRRs, pattern recognition receptors; PrtA, pneumococcal serine protease; PspA, pneumococcal surface protein A; PspK, pneumococcal surface protein K; PsrP, pneumococcal serine rich repeat protein; sIgA, secretory immunoglobulin A; SpxB, pneumococcal pyruvate oxidase; SRAs, class A macrophage scavenger receptors; StrH, beta-N-acetylgucosaminidase; Th cells, T-helper cells; TLRs, Toll-like receptors; TNF-α, tumor necrosis factor alpha
